# Serum Short-Chain Fatty Acids and Associations With Inflammation in Newly Diagnosed Patients With Multiple Sclerosis and Healthy Controls

**DOI:** 10.3389/fimmu.2021.661493

**Published:** 2021-05-06

**Authors:** Anna Olsson, Stefan Gustavsen, Thao Duy Nguyen, Margareta Nyman, Annika R. Langkilde, Tue H. Hansen, Finn Sellebjerg, Annette B. Oturai, Helle Bach Søndergaard

**Affiliations:** ^1^ Danish Multiple Sclerosis Center, Department of Neurology, Copenhagen University Hospital, Rigshospitalet, Glostrup, Denmark; ^2^ Department of Food Technology, Engineering and Nutrition, Kemicentrum, Lund University, Lund, Sweden; ^3^ Department of Radiology, Rigshospitalet, University of Copenhagen, Copenhagen, Denmark; ^4^ Novo Nordisk Foundation Center for Basic Metabolic Research, Faculty of Health and Medical Sciences, University of Copenhagen, Copenhagen, Denmark

**Keywords:** acetate, propionate, propionic acid, butyrate, SCFA, metabolomics, kynurenine pathway, neurofilament light

## Abstract

Multiple sclerosis (MS) is a chronic immune-mediated disease characterized by demyelination and neuroaxonal damage in the central nervous system. The etiology is complex and is still not fully understood. Accumulating evidence suggests that our gut microbiota and its metabolites influence the MS pathogenesis. Short-chain fatty acids (SCFAs), such as acetate, propionate and butyrate, are metabolites produced by gut microbiota through fermentation of indigestible carbohydrates. SCFAs and kynurenine metabolites have been shown to have important immunomodulatory properties, and propionate supplementation in MS patients has been associated with long-term clinical improvement. However, the underlying mechanisms of action and its importance in MS remain incompletely understood. We analyzed serum levels of SCFAs and performed targeted metabolomics in relation to biomarkers of inflammation, and clinical and MRI measures in newly diagnosed patients with relapsing-remitting MS before their first disease modifying therapy and healthy controls (HCs). We demonstrated that serum acetate levels were nominally reduced in MS patients compared with HCs. The ratios of acetate/butyrate and acetate/(propionate + butyrate) were significantly lower in MS patients in a multivariate analysis (orthogonal partial least squares discriminant analysis; OPLS-DA). The mentioned ratios and acetate levels correlated negatively with the pro-inflammatory biomarker *IFNG*, indicating an inverse relation between acetate and inflammation. In contrast, the proportion of butyrate was found higher in MS patients in the multivariate analysis, and both butyrate and valerate correlated positively with proinflammatory cytokines (*IFNG* and *TNF*), suggesting complex bidirectional regulatory properties of SCFAs. Branched SCFAs were inversely correlated with clinical disability, at a nominal significance level. Otherwise SCFAs did not correlate with clinical variables or MRI measures. There were signs of an alteration of the kynurenine pathway in MS, and butyrate was positively correlated with the immunomodulatory metabolite 3-hydroxyanthranilic acid. Other variables that influenced the separation between MS and HCs were NfL, *ARG1* and *IL1R1*, D-ribose 5-phosphate, pantothenic acid and D-glucuronic acid. In conclusion, we provide novel results in this rapidly evolving field, emphasizing the complexity of the interactions between SCFAs and inflammation; therefore, further studies are required to clarify these issues before supplementation of SCFAs can be widely recommended.

## Introduction

Multiple sclerosis (MS) is a chronic immune-mediated disease characterized by demyelination and neuroaxonal damage in the central nervous system (CNS). The etiology is complex and is still not fully understood, but both genetic and exogeneous risk factors contribute to the development of MS (1). Peripherally activated immune cells are involved in the initiation of pathogenic inflammation in the CNS and accumulating evidence suggests that our gut microbiota and intestinal epithelial barrier play a role in the immune activation in autoimmune diseases, including MS (2, 3). The gut microbiota consists of trillions of microbes that inhabit the intestinal tract and, depending on the diet, gut bacteria produce metabolites with immunomodulatory effects (4). Short-chain fatty acids (SCFAs), such as acetate, propionate and butyrate are the most abundant metabolites produced by colonic bacterial fermentation of indigestible carbohydrates and, in smaller amounts, proteins (5). Roughly 95% of the SCFAs are absorbed by the colonocytes where they either serve as a source of energy and maintain barrier integrity by regulating tight junction expression and mucus production, or they are transported to the lamina propria and further into the blood stream. Hence, only 5% are dispelled by the feces, which is why measuring SCFAs in fecal samples may be suboptimal compared with measuring circulating levels in blood (6).

SCFAs have important immunomodulatory properties mediated by inhibition of histone deacetylase (HDAC) as well as activation of their G protein-coupled receptors (GPR), such as the free fatty acid receptor 3; FFAR3 (GPR41), FFAR2 (GPR43) and hydroxycarboxylic acid receptor 2; HCAR2 (GPR109A) (7). Furthermore, SCFAs have been shown to increase the number of regulatory T cells (T_reg_) and suppress T helper (Th) 17 cells and Th1 cells, skewing the immune response towards an anti-inflammatory state (8, 9). In germ-free mice, butyrate is also able to upregulate tight junction proteins in the blood-brain barrier (BBB), thus improving the barrier integrity (10). SCFAs can furthermore cross the BBB *via* transporters located on the endothelial cells and influence neuroinflammation in the CNS (7, 11). The production of SCFAs is often reduced in dysbiotic gut microbiota, contributing to an inflammatory environment (12). Overall, animal models of MS provide convincing results that SCFAs can have an impact on the pathogenesis of experimental autoimmune encephalomyelitis (EAE) and thus likely MS. In addition, other microbiota-related metabolites, such as tryptophan and kynurenine metabolites have been shown to have immunomodulatory properties influencing several inflammatory diseases, including MS. Tryptophan can be metabolized both peripherally and in the CNS into neuroactive kynurenines and aryl hydrocarbon receptor (AHR) ligands that are able to modulate local and distant host functions involving inflammation, neurotransmission and complex immune responses. Gut microbiota are also capable of producing these neuroactive kynurenine metabolites making it interesting in relation to SCFAs (13).

Recently, a study on the effects of supplementation of propionate in MS patients was published by Duscha et al. (14) They showed that propionate supplementation in MS patients reverses the imbalance of T_reg_/Th17 cells *via* increased T_reg_ cell induction and improved T_reg_ cell function. These effects were associated with long-term clinical improvement. However, their results need to be validated in larger cohorts of newly diagnosed untreated MS patients before making actual recommendations of supplementation. Apart from that study, only a limited number of other studies have investigated SCFA levels in MS patients and with somewhat conflicting results (15–20). Moreover, these are either based on fecal analyses exclusively, or include patients on disease modifying treatment (DMT), long disease duration and/or severe disability, which might bias the results considerably. The exact effects and downstream mechanisms by which the gut microbiota and its metabolites interact with the host immune system in MS remain elusive (21). Detailed information on microbiota-related metabolites in relation to immune activation will lead to a better understanding of how the microbiota-gut-brain-axis contributes to the complex etiology and pathogenesis in MS. Our main hypothesis was that serum SCFA levels are lower in newly diagnosed MS patients before initiating their first disease modifying therapy (DMT) compared with healthy controls (HCs), and that SCFAs are associated with the gene expression of biomarkers of inflammation. Moreover, we aimed to explore possible associations between levels of SCFAs, clinical measures and metabolomics with emphasis on kynurenine pathway metabolites in newly diagnosed patients with relapsing-remitting MS (RRMS) and HCs.

## Material and Methods

### Participants

We prospectively included 58 patients with RRMS or clinically isolated syndrome (CIS) and 50 HCs, from year 2017 to 2019. Inclusion criteria were newly diagnosed (< 1 year) RRMS or CIS fulfilling the McDonald criteria 2017 (22), aged 18 to 55 years, and inclusion before initiation of the first disease modifying therapy (DMT). High-dose corticosteroid treatment was not defined as a DMT, but as a symptomatic treatment of relapses and therefore formed part of the exclusion criteria. Exclusion criteria were other autoimmune disorders, celiac disease, lactose intolerance, severe diseases (e.g. cancer, vasculitis, HIV, hepatitis), prior treatment with chemotherapy or similar medicine, pregnancy and breastfeeding, and treatment with corticosteroids or antibiotics in the last 4 weeks. HCs were healthy non-blood related family members or friends of the MS patients, aged 18 to 55 years and did not fulfil the exclusion criteria. This study is a cross-sectional study analyzing fasting peripheral blood samples from MS patients and HCs; in addition, MS patients were evaluated clinically and had magnetic resonance imaging (MRI) of the brain and spinal cord in the same 3 Tesla machine at baseline and after 12 months. Neurologic examination was performed by experienced, Neurostatus C certified neurologists, and disability was assessed by the Expanded Disability Status Scale (EDSS) (23). Disease activity was defined as the presence of gadolinium (Gd)-enhancing lesions at baseline. All participants answered an environmental and lifestyle questionnaire (GEMS – Genes and environment in MS), which was developed in Sweden and translated into Danish with permission from Karolinska Institute, Stockholm, Sweden (24). The same cohort was previously included in another study (25).

All participants signed informed consent forms. The study was approved by the regional ethics committee and Danish data protection agency (protocol no.:H-16047666).

### Assessments

Plasma tubes were immediately put on ice after sampling. The clotting time for blood intended for serum was exactly 1 hour. Serum and plasma were isolated by centrifugation at 2000 x g at 4°C for 10 minutes, aliquoted in Eppendorf polypropylene tubes and stored at -80°C. To avoid systematic bias in the analysis all measurements were performed with an even distribution of MS patients and HCs on each measured plate. We furthermore included the same internal positive control on all plates to verify that the assays were run properly.

### Single Molecule Array Assays

Serum levels of neurofilament light chain (NfL) and interleukin-6 (IL-6) were measured using a single-molecule array assay (SIMOA) (Quanterix™, NF-light and IL-6 **^®^**Advantage Kit, Billerica, MA, USA). The lower limit of quantification (LLOQ) for NfL was of 0.343 pg/ml, with a dynamic range from 0 to 1800pg/ml. For IL-6 the LLOQ was 0.0103 pg/ml with a dynamic range from 0 to 120 pg/ml. Inter- and intra-assay coefficients of variations (CVs) for assays are shown in appendix, **Table S1**.

### Gene Expression by Quantitative Real-Time PCR (qPCR)

Whole blood was sampled in PAXgene tubes, and total RNA was extracted using PAXgene Blood miRNA kit (PreAnalytix, Qiagen). One microgram total RNA was then reverse transcribed by the High Capacity cDNA Reverse Transcription kit (Life Technologies Europe B.V.). Using TaqMan technology, qPCR was performed in duplicates on 1:1 (*IL10*)), 1:10 or 1:50 diluted cDNA template with TaqMan Universal FAST PCR Master mix and target-specific primers and probes for *FFAR2, HCAR2*, aryl hydrocarbon receptor *(AHR)*, indoleamine-2,3-dioxygenase 1 *(IDO1)*, arginase-1 *(ARG1)*, interleukin-1β *(IL1B)*, IL-1 receptor type 1 *(IL1R1), IL10*, tumor necrosis factor *(TNF)*, and interferon-γ *(IFNG)* genes. PCR amplification was done on a ViiA7 real-time PCR thermal cycler (Life Technologies, USA). A pool of cDNA from 25 HCs was used in quadruplets on each PCR plate as an inter-plate calibrator. An expression index was calculated by the 2^-ΔΔCt^ method for relative quantification using GenEx Pro 6.0.5 (26). Data were normalized with the reference gene *UBE2D2* and *CASC3* since these genes are stably expressed in HCs and RRMS patients in whole blood (27). All gene expression values were set relative to the HC calibrator pool giving a gene expression index value.

### Quantification of SCFAs in Serum

We measured serum levels of acetate, propionate, butyrate and valerate along with branched SCFAs including isobutyrate and isovalerate. SCFAs were analyzed following a method by Zhao et al. (28) A polypropylene hollow fiber was immersed in diluted serum samples to extract SCFAs. The obtained SCFAs were injected onto a fused-silica capillary column with Free Fatty Acid Phase of 30 m x 0.53 mm I.D. coated with 0.50 µm film thickness (DB-FFAP 125-3237, J&W Scientific, Agilent Technologies Inc., USA) for gas chromatography analysis.

### Metabolomics

Targeted metabolomics of 102 metabolites in serum samples were analyzed by mass spectrometry-based methods at the Metabolomics Unit, Technology Centre, Institute for Molecular Medicine Finland FIMM, University of Helsinki. Briefly, final analysis for all metabolites were performed on an ACQUITY UPLC-MS/MS system (Waters Corporation, Milford, MA, USA). Chromatographic separation was done using 2.1 × 100 mm Acquity 1.7um BEH amide HILIC column (Waters Corporation, Milford, MA, USA), and temperature was maintained at 45°C. The total run time was 17.5 minutes including 2.5 minutes of equilibration step at a flow rate of 600 µL/min. For the full list of the measured metabolites and specific data on the processing of samples see appendix, **Table S2** and **Supplementary Methods**.

### Magnetic Resonance Imaging

MRI of the brain and spinal cord was performed on the same Siemens 3 T Verio scanner (Siemens, Erlangen) using a 32-channel head coil at baseline and after 12 months. MRI was performed with and without intravenous Gd-infusion (0.1 mmol/kg). The images were evaluated by the same neuroradiologist.

### Statistical Analyses

Data are presented as median (interquartile range (IQR; 25th and 75th percentiles)) or mean ± standard deviation (SD) for continuous variables, depending on normality, and frequency (percent) for categorial variables. Normality was assessed by histograms and Shapiro-Wilks test. Differences between MS and HCs were first assessed by Mann-Whitney U test. Second, data were log-transformed, where required, and one-way analysis of covariance (ANCOVA) was used to adjust for possible confounders such as sex, age, BMI, smoking (current smoker vs. non-smoker), probiotic supplementation (yes vs. no) and diet (vegetarian/vegan/pescatarian vs. omnivore). Spearman’s correlation was used to assess associations between SCFAs, metabolites, biomarkers of inflammation, and paraclinical/clinical parameters. Unless stated otherwise, p-values were corrected for multiple testing with the Benjamini-Hochberg method with a false discovery rate (FDR) significance level of 5%. Corrected p-values are reported as q-values. ANCOVA models comparing more than two groups (active vs. inactive MS and HCs) were adjusted with Bonferroni post-hoc test. Orthogonal partial least squares discriminant analysis (OPLS-DA, SIMCA version 15, Umetrics) was used to identify and visualize discriminating variables responsible for the separation patterns among MS and HCs. Data were log-transformed, and Pareto scaled before modelling to give each variable equal influence on the model. Receiver operating characteristic (ROC) curve analysis was furthermore used to evaluate the discriminative values for selected biomarkers to predict disease activity (Gd+ MRI lesions) at baseline. The index was represented graphically as the area under the curve (AUC). Two-sided *p-*values ≤ 0.05 were considered statistically significant. Statistical analyses were performed using IBM^®^ SPSS^®^ statistics (version 25). The graphs were created with GraphPad Prism^®^ 8.0, except for heatmaps that was generated using R statistical software (29).

## Results

There were no significant differences in age, sex, BMI and smoking status between MS patients and HCs. The demographic and clinical characteristics of the MS patients and HCs are shown in **Table 1**.

**Table 1:** Demographics and clinical characteristics.

	Multiple Sclerosis, n=58	Healthy controls, n=50
**Sex (female); n (%)**	44 (76%)	34 (68%)
**Age; y**	34 (27-40)	33 (28-39)
**BMI; kg/m^2^**	23 (21-26)	24 (22-26)
**Current smoker; n (%)**	9 (16%)	10 (20%)
**Supplementation of probiotics; n (%)**	5 (9%)	3 (6%)
**Vegetarian/vegan/pescatarian diet; n (%)**	8 (15%) ^a^	5 (10%)
**Disease duration (years since 1st symptom)**	0.8 (0.3-2.0)	N.A.
**EDSS**	2.0 (1.0-3.0)	N.A.
**No. of clinical relapses 1 year before baseline**	1 (1-2)	N.A.
**Presence of clinical relapses 1 year prior baseline; n (%)**	57 (98%)	N.A.
**T2 lesion count; n (%)**		
** 1-9**	16 (27.6%)	N.A
** 10-19**	9 (15.5%)	N.A
** ≥20**	33 (56.9%)	N.A
**Gd + lesion count**	0 (0-1)	N.A.
**Presence of Gd + lesions; n (%)**	21 (40%)^b^	N.A.

All data is given in median with interquartile range except if otherwise specified. BMI, body mass index; EDSS, expanded disability status scale; No., number; Gd+, gadolinium enhancing; N.A., not applicable.

a 5 patients did not answer dietary questionnaire.

b 5 patients did not receive gadolinium-infusion at baseline MRI.

### SCFAs in MS Patients *vs.* HCs

**Figure 1:**
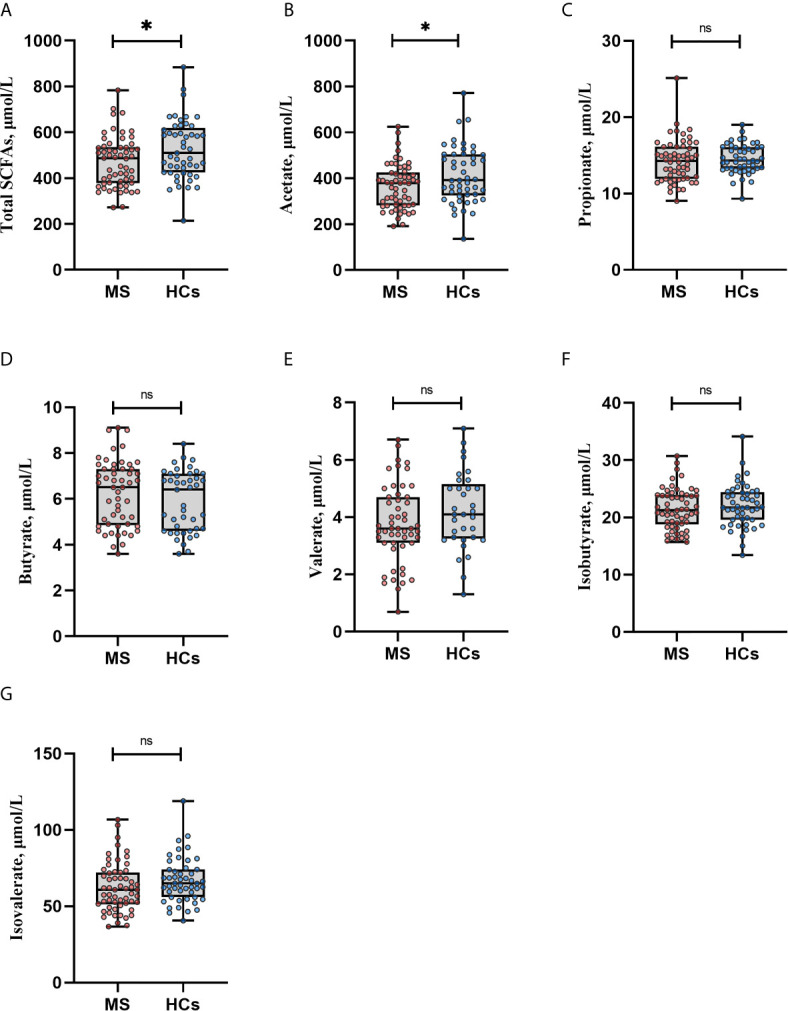
Serum SCFA levels in multiple sclerosis (MS) patients and healthy controls (HCs). **(A, B)** Total amount of short-chain fatty acids **(**SCFAs) and acetate levels were lower in MS patients compared with HCs. **(C–G)** Propionate, butyrate, valerate, isobutyrate and isovalerate did not differ significantly between MS and HCs, by Mann Whitney U test. *p<0.05. Boxplots display median, inter quartile range, minimum and maximum. Each dot represents an individual value. Similar results were obtained when adjusting for sex, age, BMI, smoking status, probiotics and dietary pattern in an ANCOVA test. However, after adjustment for multiple testing all q values became non-significant, q>0.05. SCFAs: short-chain fatty acids, ns., non-significant.

The total concentrations of SCFAs were lower in MS patients compared with HCs (453 μmol/L vs. 510 μmol/L, p=0.022), this was predominantly carried by acetate, which was the only SCFA that was significantly lower in MS patients compared with HCs (349 μmol/L vs. 402 μmol/L, p=0.021) (**Figures 1A, B**). However, after FDR correction q-values became non-significant, q=0.067. There were no statistically significant differences in the levels of propionate, butyrate, valerate, isovalerate or isobutyrate (**Figures 1C–G**). All analyses were adjusted for age, sex, BMI, smoking, dietary pattern (vegetarian/vegan/pescatarian vs. omnivore) and probiotics use. None of the patients received corticosteroids the last four weeks before baseline as this was an exclusion criterion. To exclude a possible interference of previous high-dose corticosteroid treatment we compared SCFAs levels between patients that had been treated with corticosteroids in the period of >4 to 12 weeks prior inclusion (n=22, 38%) and those that had not (n=36, 62%). There were no differences in SCFA levels between the groups (data not shown).

### SCFAs – Associations With Biomarkers of Inflammation and GPRs

**Figure 2:**
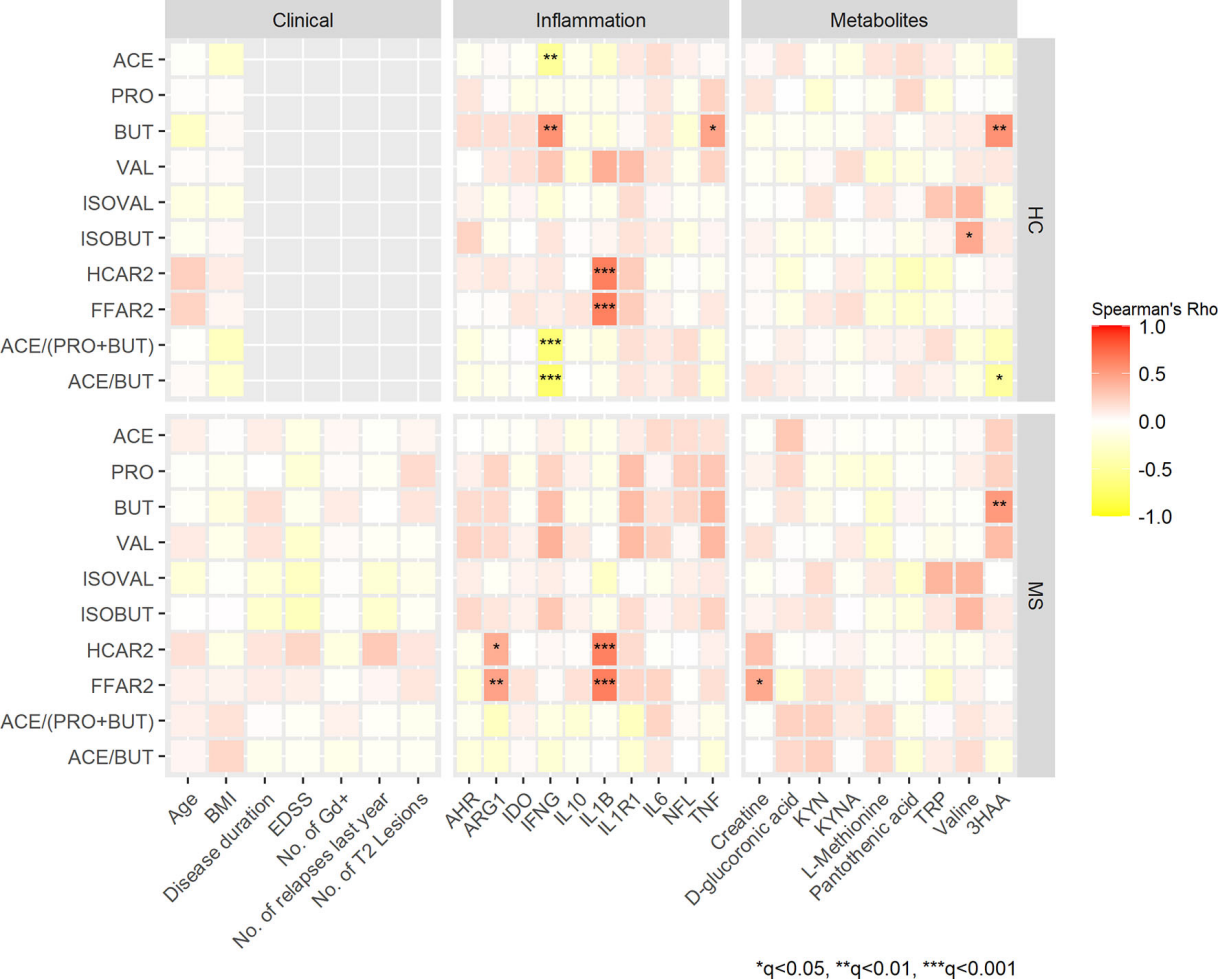
Spearman’s correlation heatmap of the associations between SCFAs, clinical variables, biomarkers of inflammation and selected metabolites in patients with MS and HCs. Color gradients indicate rho values; positive correlations are marked in red, negative in yellow. Clinical measures that were not applicable for HCs are in grey. *q<0.05, **q<0.01 and ***q<0.001. ACE, acetate; PRO, propionate; BUT, butyrate; VAL. valerate; IVAL, isovalerate; IBUT; isobutyrate. HCAR2, hydroxycarboxylic acid receptor 2; FFAR2, free fatty acid receptor 2; BMI, body mass index; EDSS, expanded disability status scale, No. of Gd+, number of gadolinium enhancing lesions; AHR, aryl hydrocarbon receptor ARG1, arginase 1; IDO1, indoleamine 2,3-dioxygenase 1; IFNG, interferon-gamma; IL, interleukin; IL1B, IL-1 beta; IL1R1, IL-1 receptor type 1; NFL, neurofilament light chain; TNF, tumor necrosis factor; KYN, kynurenine; KYNA, kynurenic acid; TRP, tryptophan; 3HAA, 3-hydroxyanthranilic acid.

To examine whether levels of SCFAs were associated with biomarkers of inflammation, we performed correlation analyses. In HCs, acetate concentrations in serum correlated inversely with the expression of the pro-inflammatory cytokine *IFNG* (rho= -0.524, q<0.01), whereas butyrate concentrations correlated positively with *IFNG* (rho=0.559, q<0.001) and *TNF* (rho=0.468, q<0.05) (**Figure 2**). In MS patients, none of these correlations survived the FDR correction, but butyrate and valerate concentrations showed nominally significant positive correlations with *TNF* (rho=0.374, p=0.006 and rho=0.370, p=0.006, respectively). Furthermore, valerate also correlated positively with *IFNG* (rho=0.397, p=0.003) and *IL1R1* (rho=0.356, p=0.009) (**Appendix, Figure S1**).

SCFAs are known to mediate some of their immunomodulating properties by activating GPRs on immune cells. Thus, we looked for possible correlations between GPRs and biomarkers of inflammation. We observed strong positive correlations of both *FFAR2* and *HCAR2* with the pro-inflammatory cytokine *IL1B* in MS patients (rho=0.638 and rho=0.647, q<0.001) and in HCs (rho=0.643 and rho=0.638, q<0.001). Furthermore, *FFAR2* and *HCAR2* correlated with *ARG1* in MS patients (rho=0.423 and 0.478, q<0.01, q<0.05, respectively). None of the SCFAs or the expression of their receptors correlated significantly with NfL levels in MS patients or HCs (**Figure 2**). Significant correlations between SCFAs and the gene expression of their receptors were found in HCs. Acetate levels correlated negatively with *HCAR2* (rho= -0.292, p= 0.042) and *FFAR2* (rho= -0.312, p=0.029), while valerate correlated positively with *HCAR2* (rho=0.442, p=0.010). In MS patients none of the SCFAs correlated significantly with the gene expression of their receptors (data not shown).

### Ratios of SCFAs

To minimize the possible influence of conversion between the SCFAs, their uptake by peripheral organs and alterations during sample processing we analyzed ratios and proportions (%) of the SCFAs. The ratios of acetate/butyrate and acetate/(propionate + butyrate) were lower in MS patients compared with HCs (49 *vs*. 65, p=0.005 and 16 *vs*. 19, p=0.010). After FDR correction q-values were 0.06 for both ratios. Next, we analyzed the proportions of single SCFAs in relation to the total amount of SCFAs, but no significant differences between MS and HCs were detected when adjusting for confounding factors. However, a higher proportion of butyrate was found in subjects taking probiotics, while higher proportions of acetate was found in the group of vegetarians/vegans/pescatarians (data not shown). Finally, we performed Spearman’s correlation analyses revealing negative correlations between *IFNG* and the ratios of acetate/butyrate and acetate/(propionate + butyrate) (rho= -0.689 and rho= -0.735, q<0.001 in HCs (**Figure 2**). In MS patients, only a nominally significant correlation was found between *IL1R1* and the ratio of acetate/(propionate + butyrate) (rho= -0.320, p=0.016) (Appendix, **Figure S1**). Scatter plots of selected correlations with q<0.01 and rho-values stronger than 0.5 are presented in **Supplementary Figure S2**.

### Metabolomics, AHR and IDO-1

**Figure 3:**
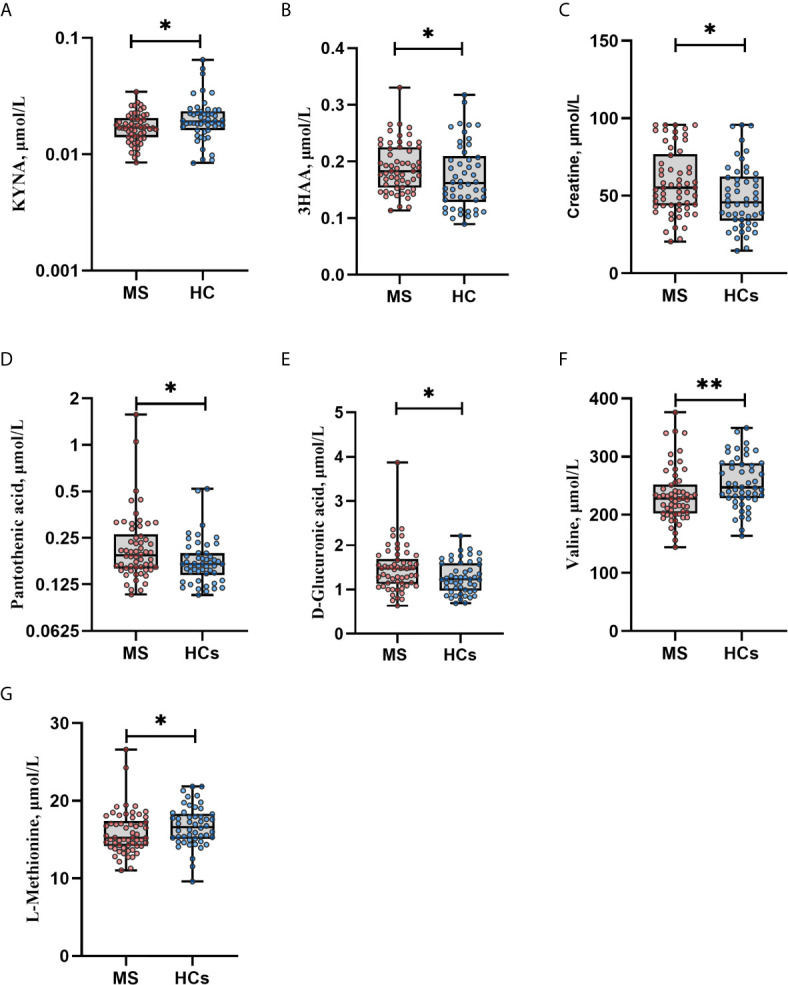
Metabolites in multiple sclerosis (MS) *vs*. healthy controls (HCs). **(A)** KYNA levels were lower in MS *vs*. HCs. **(B)** 3HAA levels were higher in MS *vs*. HCs. **(C–E)** MS patients had higher levels of creatine, pantothenic acid and D-glucuronic acid compared with HCs. **(F–G)** Levels of valine and l-methionine were lower in MS patients vs. HCs. *p<0.05, **p<0.01 by Mann Whitney U test. Data are median (IQR), minimum and maximum. Each dot represents individual values. After adjustment for multiple testing all q values became non-significant, q>0.05. Due to right-skewed distribution, logarithmic scales were used to display KYNA and pantothenic acid levels. KYNA, kynurenic acid; 3HAA, 3-hydroxyanthranilic acid.

SCFAs might be related to other neuroactive metabolites, and therefore we performed targeted metabolomics with emphasis on tryptophan and kynurenine pathway metabolites: tryptophan, L-kynurenine, kynurenic acid (KYNA), 3-hydroxy-dl-kynurenine (below limit of quantification; BLOQ), 3-hydroxyanthranilic acid (3HAA) and nicotinamide adenine dinucleotide (NAD) (BLOQ). Seven of 79 quantified metabolites (**Table S2**) differed nominally between MS patients and HCs with Mann-Whitney U test. MS patients had higher levels of creatine (median: 55 *vs*. 46 μmol/L, p=0.022), pantothenic acid (vitamin B5) (0.19 *vs*. 0.17 μmol/L, p=0.021) and D-glucuronic acid (1.5 vs. 1.2 μmol/L, p=0.031). Levels of valine and l-methionine were lower in MS patients compared with HCs (228 *vs*. 247 μmol/L, p=0.005 and 15 vs. 17 μmol/L, p=0.025 for valine and methionine, respectively). Regarding the kynurenine pathway, levels of KYNA were lower in MS patients (0.017 *vs*. 0.019 μmol/L, p=0.019) and 3HAA levels were higher (0.18 *vs*. 0.16 μmol/L, p=0.040). After adjustment for multiple tests, all q-values were >0.05 (**Figure 3**). Twenty-three metabolites were not detected due to concentrations below the limit of quantification or were excluded from analyses during quality control. Furthermore, we analyzed the mRNA expression of *IDO1* that is known to be the rate limiting enzyme in the kynurenine pathway, and *AHR* that is a ligand-activated transcription factor for which *IDO1* acts as an agonist and thereby provides feedback loop. However, there were no differences in *IDO1* or *AHR* expressions between MS patients and HCs (data not shown). We also investigated the possible connections between SCFAs and the metabolites that were differentially expressed at a nominal significance level, as well as the kynurenine metabolites. In both HCs and MS patients, 3HAA correlated positively with butyrate (rho=0.556, q<0.001 and rho=0.517, q<0.001, respectively) (**Figure 2**).

### SCFAs – Associations With Clinical Variables and MRI Measures

The branched SCFAs isobutyrate and isovalerate were nominally correlated with disability assessed by EDSS (rho= -0.341, p=0.009, rho= -0.308, p=0.019, respectively), none of which was significant with adjustment for multiple comparisons (**Figure S3**). Otherwise SCFAs did not correlate with clinical variables or MRI measures (**Figure 2**). There were no significant differences in levels of SCFAs between patients with (n=42, 72%) or without (n=12, 21%) spinal lesions on MRI. Four patients did not have a complete spinal cord MRI (data not shown). There were no significant correlations between the time since last clinical relapse and SCFA levels. The median time since last relapse was 3.5 months (IQR: 2-6 months).

### SCFAs and 12 Months Follow-Up

Twenty-two patients (38%) developed new or enlarging T2 lesions at 12 months follow up. This group of patients had nominally higher levels of propionate (13.5%, CI 95%: 2 - 26%, p=0.026) and valerate (18%, CI 95%: 11 – 35%, p=0.037) compared with stabile patients without new or enlarging lesions, but these results were not significant after FDR correction, q=0.15 for both. New clinical relapses were experienced by seventeen patients (30%) but SCFAs levels did not differ significantly between patients with and without clinical relapses at month 12 (data not shown).

### Active *vs*. Inactive MS Patients, Compared With HCs

To explore whether SCFAs or other metabolites were associated with disease activity, we stratified patients based on whether they presented Gd+ lesions on MRI at inclusion (*active*) or not (*inactive*). There were no statistically significant differences when comparing active MS with inactive MS. However, levels of acetate, valine and KYNA were lower in inactive MS patients compared with HCs, when adjusting for confounders and multiple comparisons (data not shown). ROC curve analyses were not able to identify possible biomarkers of disease activity regarding the SCFAs and metabolites. AUC was less than 0.65 for all the mentioned metabolites (data not shown).

### OPLS-DA of SCFAs, Metabolites and Biomarkers of Inflammation

**Figure 4:**
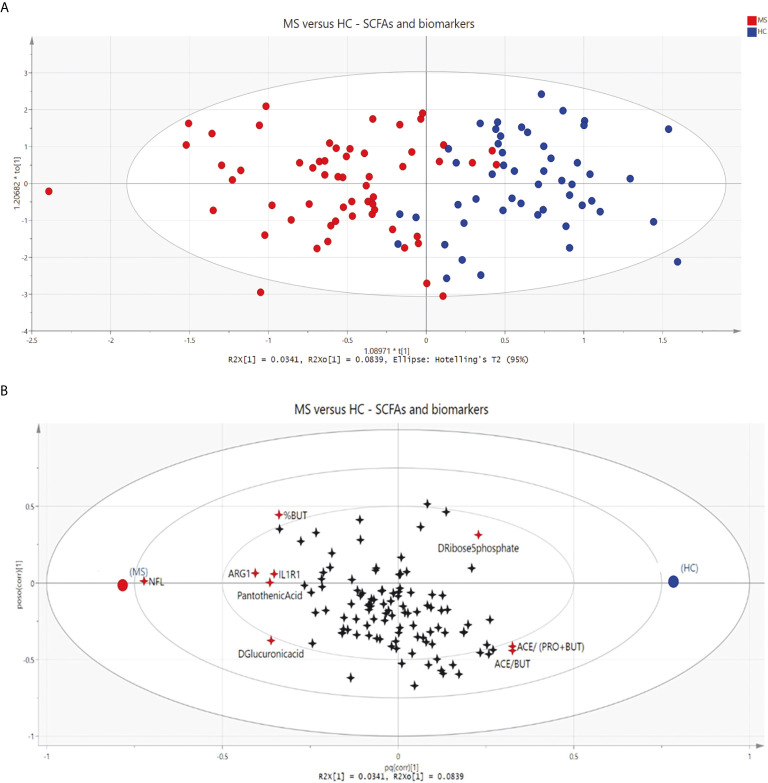
**(A)** Score scatter plot displaying group separation between patients with multiple sclerosis (MS, red circles) and healthy controls (HCs, blue circles) based on short-chain fatty acids; biomarkers of inflammation and metabolites. Each circle represents one participant. **(B)** Loading scatter plot showing changes in short-chain fatty acids (SCFAs), biomarkers of inflammation and metabolites. Groups are shown as circles (MS, red; HCs, blue), and variables as 4-point stars. Discriminating variables are marked in red. NFL, neurofilament light chain; ARG1, arginase 1; IL1R1, interleukin-1 receptor type 1; %BUT, proportion of butyrate; ACE/(PRO+BUT), acetate/(propionate + butyrate); ACE/BUT, acetate/butyrate.

A multivariate data analysis was conducted to elucidate how SCFAs, metabolites and biomarkers of inflammation were distributed and related to each other in MS patients and HCs (**Figures 4A, B**). The OPLS-DA model accounts for 11.8% of the variation of the included variables and 61.4% of this variation is related to the class separation membership, indicating that the model fits the data as evidenced by an almost complete separation between MS patients and HCs (**Figure 4A**). Despite low predictivity (Q^2^<0.5), the model was significant by cross-validation. Furthermore, 8.39% of the data variation was common within groups and uncorrelated to class separation (**Figure S4**). Serum NfL was the strongest discriminating variable, followed by *ARG1* and the ratio of acetate/butyrate. Other variables that also influenced the separation were acetate/(propionate + butyrate), D-ribose 5-phosphate, proportion of butyrate (%BUT), *IL1R1*, pantothenic acid and D-Glucuronic acid (**Figure 4B**).

## Discussion

In this study we measured serum levels of SCFAs and biomarkers of inflammation, and performed targeted metabolomics in blood samples of newly diagnosed RRMS patients before their first DMT, and HCs to better understand how the microbiota-gut-brain-axis contributes to the complex etiology and pathogenesis of MS. We found that serum acetate levels were nominally reduced in MS patients compared with HCs. Furthermore, these levels correlated negatively with IFN-γ in HCs, indicating an inverse relation with inflammation. Acetate is the most abundant SCFA produced by gut bacteria but can also be derived by acetyl-CoA from glycolysis; moreover, acetate can be transformed into butyrate by a cross-feeding mechanism by some bacterial strains in the colon (7). Serum acetate has previously been reported to be lower in MS patients compared with HCs in a study on secondary progressive MS (SPMS) patients, which indicates another microbiota composition (17). In contrast, a recent study on RRMS patients reported higher levels of acetate in plasma samples, compared with HCs (19). However, this was based on stratified EDSS levels showing statistically significant differences only between patients with a relatively high disability (EDSS≥5.0) compared with low disability (EDSS≥1.5) and HCs. No significant differences were seen between patients with low disability and HCs. Furthermore, it was not specified if the participants were fasting at blood sampling, which is critical for the evaluation of SCFAs. Shortly after a meal containing dietary fiber SCFAs levels can be drastically altered (30). In our study, the patients had a much lower median EDSS of 2.0 (1.0-3.0) at inclusion, and all patients and HCs were fasting at blood sampling, therefore the results are not entirely comparable. Additionally, data on BMI, diet, smoking status and probiotics was recorded in our study and adjusted for in the statistically analyses due to the possible interference with gut microbiota composition and the following SCFA production (31–34).

The most important negative finding in our study was that serum levels of propionate were not lower in patients with MS compared with HCs. This was in contrast with our hypothesis and furthermore in contrast with the results of a recently published paper (14). A reduction of propionate in both serum and stool samples of MS patients was shown to be associated with altered gut microbiota and an imbalance of T_reg/_Th17 cells, which was reversed by oral supplementation of propionate (14). They did not report significant differences for acetate or butyrate, but the study involves several steps validating their findings for propionate. However, of the roughly 300 patients included in different analyses, only a smaller cohort of 36 MS patients were newly diagnosed and treatment naïve. The study had power to detect differences in small sample sizes; nevertheless, confounding by treatment in the group of patients receiving DMT cannot be entirely ruled out. Although blood samples were taken in the morning hours, MS patients and HCs were not fasting, which might have influenced their results. Another study found reduced serum levels of butyrate in MS patients and higher levels of caproic acid, a medium-chain fatty acid, compared with HCs. They did, however, not report whether the participants were fasting at blood sampling (15). Concurrently, they found that microbiota was depleted of butyrate producing bacteria and enriched in pro-inflammatory species. Butyrate was furthermore inversely correlated with plasma levels of lipopolysaccharide (LPS) and intestinal fatty acid-binding protein (I-FABP), suggesting that the butyrate reduction was responsible for the increased intestinal barrier permeability that has previously been described in MS patients (35, 36). Our results did not confirm the reduced butyrate levels in MS patients. In contrast, our multivariate analysis showed that the proportions of butyrate tended to be higher in MS patients compared with HCs.

Accumulating evidence for the overall protective effect of SCFAs in EAE models and latest in MS patients have somewhat diminished the attention towards the possibility of bidirectional regulatory potentials of SCFAs (9). Studies have shown that SCFAs can promote T-cell differentiation directly into T cells producing pro-inflammatory cytokines such as IL-17 and IFN-gamma, and/or anti-inflammatory IL-10 depending on the cytokine context (17, 37). In our study, butyrate and valerate were nominally associated with pro-inflammatory biomarkers both in MS patients and HCs. Importantly, butyrate has been shown to induce inflammatory responses by activating FFAR3 and FFAR2 depending on the different cell types and the cytokine milieu (38, 39). Furthermore, Ffar3^-/-^ and Ffar2^-/-^ mice have been shown to be more resistant to EAE pathogenesis than wild type mice (17). This is interesting, since we observed relatively strong positive correlations in both MS patients and HCs of *FFAR2* and *HCAR2* with the expression of *IL1B*.

Of interest, *FFAR2* and *HCAR2* also correlated with the expression of *ARG1* in MS patients. *ARG1* codes for arginase-1, an enzyme present in immuno-regulatory macrophages, which may contribute to modulation of inflammation in EAE, leading to recovery of EAE paralysis (40). These findings highlight the complex functions of SCFAs and their receptors in the regulation of autoimmune inflammation in the CNS. However, only acetate and valerate showed significant correlations with the gene expression of their receptors in HCs, this was not seen in MS patients and might partly be explained by the limited sample size. Since we analyzed mRNA gene expression in whole blood it was not possible to determine exactly from which cell subsets these genes derive, which might have concealed some associations. Furthermore, the investigated GPRs can have other ligands beyond SCFAs, which also provides an explanation why these receptors may have different immunomodulatory properties than the SCFAs (41).

In our study, the concentration of acetate, propionate and butyrate were higher compared with previous studies (14, 19, 42). This might be explained by the use of different extraction methods for the detection of SCFAs between the studies. The hollow fiber supported membrane liquid extraction (followed by GC-FID analysis) that was used in our study was developed as a technique for preconcentration and clean up to be able to quantify six SCFAs (in most methodologies in serum at most three SCFAs are analyzed). The preconcentration step is an efficiently precise key factor (28). However, it cannot be excluded that differences in SCFA profile were influenced by factors such as specific stages of disease, diet/substrate availability, gut microbiota composition and transit time.

The branched SCFAs were nominally correlated with EDSS, suggesting lower disability with higher levels of isobutyrate and isovalerate. Today, relatively little is known about branched SCFAs. They are produced in small amounts in the intestinal tract by proteolytic fermentation. Nevertheless, they relate to the other SCFAs due to cross-feeding (12, 43). However, no other convincing associations between clinical variables and SCFAs were observed. A possible reason for this might be that some of the MS patients immediately improve their lifestyle in order to alleviate the disease, when getting diagnosed. This might lead to a diet with higher amounts of vegetables containing dietary fiber, resulting in a beneficial alteration of microbiota with an increased SCFA production; hence, covering true correlations. However, we tried to minimize this by adjusting for dietary pattern. Surprisingly, patients that developed new or enlarging T2 lesions during the first year of follow-up seemed to have nominally higher fasting levels of propionate and valerate at baseline. This might be explained by dietary changes in relation to having been diagnosed with MS prior to the baseline visit or possibly by longer transit time due to MS related bowel symptoms, which also may contribute to higher levels of SCFAs.

An increasing amount of evidence supports the involvement of kynurenine metabolites and antigen presenting cells expressing *IDO1* in neuroinflammatory diseases such as MS (13). In this study, we found nominally lower levels of KYNA and higher levels of 3HAA in MS patients. However, in the multivariate analysis the kynurenine metabolites were not detected as crucial discriminating factors between MS and HCs. Animal studies indicate that both KYNA and 3HAA can ameliorate autoimmunity by suppressing the activity of Th1 and Th17 cells, inhibit production of pro-inflammatory cytokines and increase T_reg_ response in EAE models (44, 45). However, studies on MS patients reveal conflicting results (13). In general, there seems to be a short-term benefit of activation of the kynurenic pathway as a response to an increased inflammatory state, hence creating an immunosuppressant feed-back loop. In contrast, chronic activation of the kynurenic pathway may induce neurotoxic metabolites inhibiting remyelination and innate repair mechanisms (46).

The multivariate analysis visualizes discriminating variables responsible for the separation patterns among MS and HCs. However, the model might have overfitted the data and we did not have an independent validation cohort to control for this. Therefore, the results from the OPLS-DA model should be interpreted carefully and the model should be considered a complementary tool to confirm the results from univariate analyses and to get an overall visualization of the data. Serum NfL was the strongest discriminating variable between MS and HCs. NfL is an axonal cytoskeleton protein exclusively found in neurons and is released to the CNS and subsequently to the blood upon neuroaxonal damage (47). The discriminating properties of NfL were expected due to the strong associations with neuroaxonal damage, which is a hallmark of MS pathogenesis (48, 49). NfL levels were not associated with SCFAs or their receptors, which suggest that SCFAs do not directly influence neuroaxonal damage, and that there probably is a more complex association between theses variables. *ARG1* and *IL1R1* were other biomarkers of inflammation that seemed to influence the separation of MS and HCs. *IL1R1* is the receptor for the pro-inflammatory cytokine IL-1, which is known to be involved in the MS pathogenesis (50). Compared with SCFAs and the other metabolites these biomarkers of inflammation probably reflect a less complex association with the inflammatory processes seen in MS patients. Other variables that also influenced the separation between MS patients and HCs were the ratios of SCFAs; acetate/butyrate and acetate/(propionate + butyrate), and the proportion of butyrate. In contrast, actual acetate levels or the total amount of SCFAs did not turn out to be discriminating factors in the multivariate model, which makes it interesting to further investigate the implications of ratios of SCFAs in future studies. Addressing ratios might minimize the influence of cross-feeding or interconversion between SCFAs in the intestines. Cross-feeding seems essential for the balance of the actual colon SCFA levels and might therefore indirectly influence levels in serum, although to a lesser extent. Cross-feeding can be exemplified by the mechanism of some bacterial strains using acetate to produce butyrate and to a lesser extent butyrate used for production of propionate (12). Negative correlations between pro-inflammatory factors (*IFNG* and *IL1R1)* and the ratios of acetate/butyrate and acetate/(propionate + butyrate) indicate an inverse relation between these ratios and inflammation.

Three other metabolites also contributed to the discrimination between MS patients and HCs. Pantothenic acid and D-glucuronic acid levels were higher in MS patients than in HCs, and D-ribose 5-phosphate levels were lower in MS patients. D-ribose 5-phophate and SCFAs are metabolites of the pentose phosphate pathway (PPP), which is known to protect cells from oxygen radical damage (51). D-ribose 5-phophate and the ratios of SCFAs were located on the same side in the multivariate model, potentially indicating an altered PPP in MS. Glucuronic acid has been implicated in inflammatory responses by acting on toll-like receptor 4 and inducing pain in rats (52). However, relatively little is known about the effects of these metabolites in MS and further clarification of the underlying molecular mechanisms of these metabolites in the MS pathogenesis are warranted.

Strengths of this study are the clinical evaluation and blood sampling of the patients that were performed by only two physicians. MRI was evaluated on the same 3T MR machine and evaluated by the same neuroradiologist. MS patients were newly diagnosed and included before they received their first DMT. All patients and HCs were fasting at blood sampling. Furthermore, we were able to adjust for several possible confounding factors such as smoking, BMI, dietary patterns and probiotics. However, some limitations should be addressed. Being a cross-sectional study, we cannot imply causality. Long term follow-up data are required to clarify the complex interplay between the kynurenines, SCFAs and inflammation in MS. We cannot rule out that the sample size was too small to detect significant differences of the metabolomics data and we did not have a second independent cohort to validate our findings, which is an important limitation as some results were not statistically significant after adjustment for multiple testing.

In conclusion, we found that acetate levels were nominally lower in MS patients compared with HCs. Furthermore, the ratios of acetate/butyrate and acetate/(propionate + butyrate) were significantly lower in MS patients vs. HCs in the multivariate model. The mentioned ratios correlated negatively with pro-inflammatory biomarkers, indicating an inverse relation between acetate levels and inflammation. Branched SCFAs were nominally associated with clinical disability, which provides hypotheses that can be examined in future studies. There were signs of an alteration of the kynurenine pathway in our MS patients, and butyrate was positively correlated with the immunomodulatory metabolite 3HAA. However, we were not able to validate previous findings of reduced levels of propionate and butyrate reported by two other groups. In contrast, the proportion of butyrate was found higher in MS patients in a multivariate analysis, and both butyrate and valerate correlated positively with pro-inflammatory cytokines, suggesting complex bidirectional regulatory properties of SCFAs. The present study is one of the first to analyze associations between fasting serum levels of SCFAs in relation to kynurenine pathway metabolites, biomarkers of inflammation and clinical measures in newly diagnosed MS patients. We provide novel results in this rapidly evolving field, emphasizing the complexity of the interactions between SCFAs and inflammation; therefore, further studies are required to clarify these issues before supplementation of SCFAs can be widely recommended.

## Data Availability Statement

The study data are unsuitable for public deposition due to ethical restrictions and privacy of participant data. Data are available for any interested researcher who meets the criteria for access to confidential data. The corresponding author may be contacted to request study data.

## Ethics Statement 

The studies involving human participants were reviewed and approved by De Videnskabsetiske Komiteer for Region Hovedstaden (protocol no.:H-16047666). The patients/participants provided their written informed consent to participate in this study. 

## Author Contributions

Conceptualization and methodology: AO, FS, ABO, HBS. Data collection: AO, SG. Evaluation of MRI data: ARL. Data acquisition (SIMOA, qPCR): HBS. Investigation and formal analyses: AO, Analysis of SCFAs, multivariate statistical model: TDN. Results interpretation and discussion: AO, THH, TDN, MN, ABO, HBS. Supervision: HBS, ABO, FS. Original draft preparation: AO. Writing - reviewing and editing: AO, SG, TDN, MN, AL, THH, FS, ABO, HBS. All authors contributed to the article and approved the submitted version.

## Funding

This research was supported by the Danish Multiple Sclerosis Society [A-38469 and A35043], Aase og Ejnar Danielsens Fond [10-002094], Overlæge Johan Boserup og Lise Boserups Legat [J.nr. 20795-24], Grosserer A.V. Lykfeldt og Hustrus Legat, Trigon fonden, Civilingeniør Bent Bøgh og hustru Inge Bøghs fond, Carl og Ellen Hertz Legat [J.nr. 7179-2], Direktør Emil C. Hertz og hustru Inger Hertz’ Fond, Civilingeniør Frode V. Nyegaard og Hustrus Fond, Th. Maigaards Eftf. Fru Lily Benthine Lunds Fond, Jascha Fonden, Torben og Alice Frimodts Fond. The funders did not contribute to the study design, execution, analyses, interpretation of data, or writing.

## Conflict of Interest

AO has received support for congress participation from Roche and Novartis. SG has received support for congress participation from Merck. FS has served on scientific advisory boards for, served as consultant for, received support for congress participation or received speaker honoraria from Alexion, Biogen, Merck, Novartis, Roche and Sanofi Genzyme. His laboratory has received research support from Biogen, Merck, Novartis, Roche and Sanofi Genzyme. ABO has served on scientific advisory boards for Biogen Idec, Novartis and Sanofi Genzyme; has received research support from Novartis; has received speaker honoraria from Biogen Idec, Novartis and TEVA; and has received support for congress participation from, Merck, TEVA, Biogen, Roche, Novartis and Sanofi Genzyme.

The remaining authors declare that the research was conducted in the absence of any commercial or financial relationships that could be construed as a potential conflict of interest.
