# Supplementary material for: Serum Short-Chain Fatty Acids and Associations With Inflammation in Newly Diagnosed Patients With Multiple Sclerosis and Healthy Controls
Source: Front Immunol. 2021 May 6;12:661493. doi: 10.3389/fimmu.2021.661493 (PMC8134701; doi:10.3389/fimmu.2021.661493)
Supplement: Supplementary file 1 [file DataSheet_1.pdf]

Appendix

Table of contents

Table S1. Inter- and intra-assay coefficients of variations (CVs) for SIMOA

Table S2. Full list of the measured metabolites

Figure S1. Heatmap of nominally significant correlations between SCFAs, clinical measures, biomarkers of inflammation and metabolites in MS and HCs

Figure S2. Scatter plots of selected correlations

Figure S3. Scatter plots of EDSS and the branched SCFAs

Figure S4. Model window of the OPLS-DA model

Supplementary methods. Procedure of metabolomic analyses

Table S1. Inter- and intra-assay coefficients of variations for SIMOA

	Inter-assay CV (%)	Intra-assay CV (%)
**IL-6 low**	11.5	1.4
**IL-6 high**	15.0	5.2
**NfL low**	13.4	9.8
**NfL high**	4.7	2.7

Mean inter- and intra-assay coefficients of variations (CVs) of high and low controls of NfL and IL-6 measured by SIMOA. Data are presented in percentage (%).
NfL: neurofilament light chain, IL-6: interleukin-6

Table S2. Full list of the measured metabolites

S.NO	Metabolite Name	S.NO	Metabolite Name
	**1. Amino acids and derivatives**		**8. Nucleobases**
1	2-Aminoadipic Acid	52	Adenine (BLOQ)
2	3-OH-Anthranilic Acid	53	Cytosine (Mostly BLOQ)
3	3-OH-DL-Kynurenine (BLOQ)	54	Hypoxanthine
4	Alanine	55	Neopterin
5	2-Aminoisobutyric acid	56	Orotic acid (CC not ok)
6	Arginine	57	Uracil
7	Asparagine	58	Xanthine
8	Aspartic acid		**9. Neurotransmitter metabolic intermediates**
9	Creatine	59	GABA
10	Creatinine	60	5-Hydroxyindole-3-acetic acid
11	Cystathionine	61	L-5-Hydroxytryptophan
12	Dimethyl Glycine	62	Normetanephrine
13	Glutamic Acid		**10. Nucleosides**
14	Glutamine	63	2'-deoxycytidine
15	Glycine	64	2'-deoxyuridine
16	Guanidinoacetic Acid	65	Adenosine
17	Histidine	66	Cytidine
18	Homocysteine (Mostly BLOQ)	67	Guanosine
19	Homogentisic acid	68	Inosine
20	Homoserine (CC not ok)	69	Xanthosine
21	Hydroxyproline		**11. Organic compounds**
22	Isoleucine	70	D9 Trimethylamine-N-Oxide
23	Kynurenic Acid	71	1-methylhistamine
24	Kynurenine	72	Glucuronate
25	Leucine	73	Hippuric acid (Mostly BLOQ)
26	Lysine		**12. Choline mtabolites/Others**
27	Methionine	74	Allantoin
28	Phenylalanine	75	Betaine
29	Proline	76	Carnitine
30	Symmetrical DiMethylArginine (SDMA)	77	Carnosine
31	Asymmetrical DiMethylArginine (ADMA)	78	Choline
32	Serine	79	Cotinine (Mostly BLOQ)
33	Threonine	80	Gamma-Glutamylcysteine (Mostly BLOQ)
34	Tryptophan	81	Spermidine (BLOQ)
35	Tyrosine		**13. Carbohydrates**
36	Valine	82	Sorbitol (PCH)
	**2. Bile Acids**	83	UDP-Glucose (BLOQ)
37	Chenodeoxycholic Acid (Mostly ULOQ)	84	Myo inositol (CC not OK)

38	Cholic Acid (Mostly ULOQ)	85	Sucrose (PCH)
39	Glycocholic Acid		**14. Urea cycle intermediates**
40	Taurine	86	Citrulline
41	Taurochenodeoxycholic Acid	87	Ornithine
42	Taurocholic Acid		**15. Acylcarnitines**
	**3. Central Carbon Metabolites**	88	Acetylcarnitine
43	Glyceraldehyde	89	Propionylcarnitine
44	Ribose-5-Phosphate	90	Isobutyryl Carnitine
	**4. Cholesterol & Steroid metabolicintermediates**	91	Isovaleryl Carnitine
45	Acetoacetic acid (Ketone body)	92	Hexanoylcarnitine
	**5. Nucleotides**	93	Octanoylcarnitine
46	AMP (BLOQ)	94	Decanoylcarnitine
47	cAMP		**16. Enzyme Cofactors**
48	cGMP (BLOQ)	95	4-Pyridoxic Acid (B6)
49	IMP	96	Folic Acid (B9) (Mostly BLOQ)
	**6. TCA cycle intermediates**	97	Glutathione (reduced) (Mostly BLOQ)
50	Succinate	98	NAD (BLOQ)
	**7. Ethanolamines**	99	Niacinamide (B3)
51	Phosphoethanolamine	100	Nicotinic Acid (B3) (BLOQ)
		101	Pantothenic Acid (B5)
		102	Pyridoxine (B6) (BLOQ)

Full list of the measured metabolites. BLOQ; below limit of quantification, CC not ok: calibration curve not ok, ULOQ; upper limit of quantification, PCH; poor chromatography, Mostly BLOQ or mostly ULOQ means only few samples had concentrations within calibration range, and therefore these metabolites were excluded from analyses. S.NO: sample number

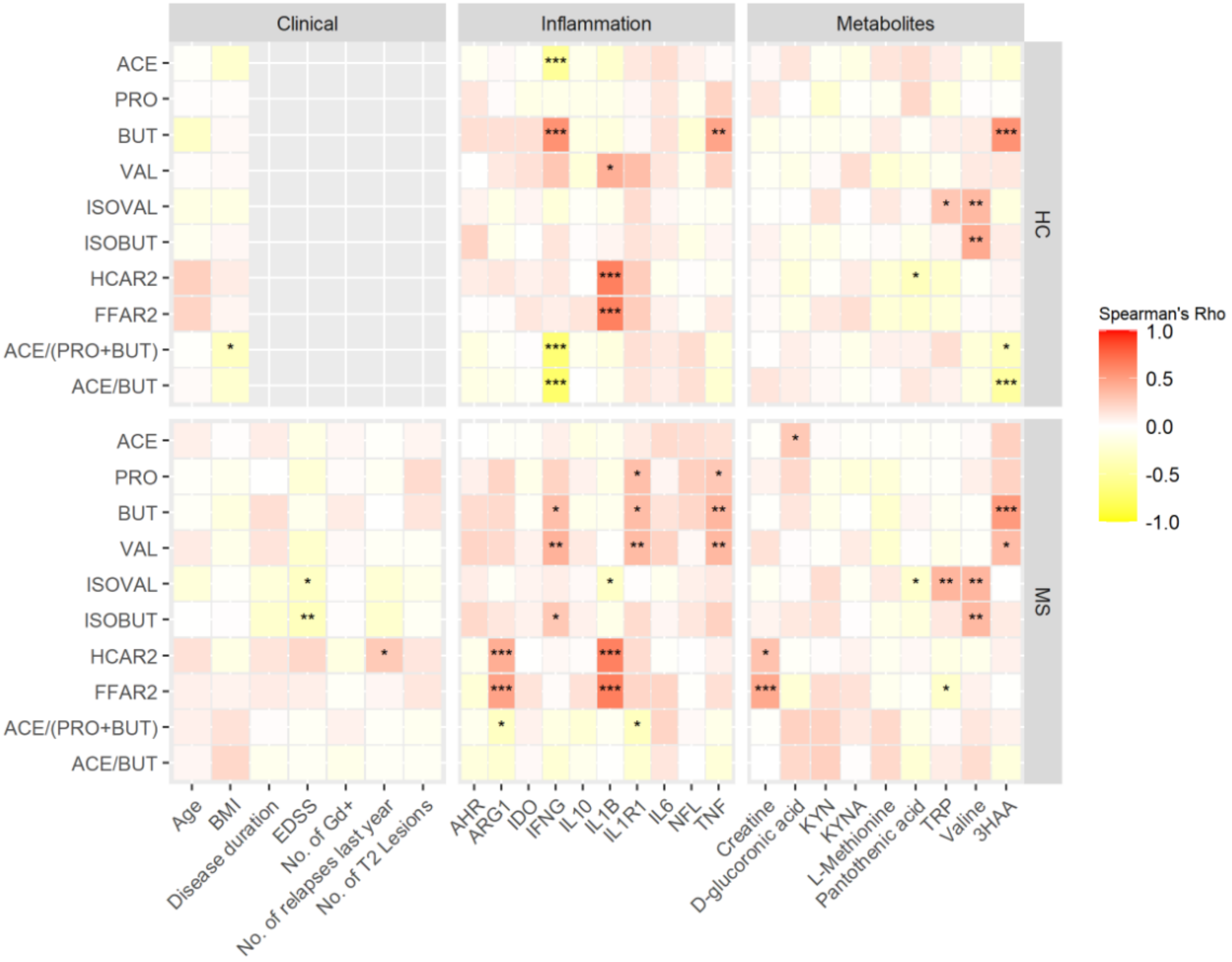

Figure S1. Unadjusted Spearman's correlation heatmap of the associations between SCFAs, clinical variables, biomarkers of inflammation and selected metabolites in patients with MS and HCs. Color gradients indicate rho-values; positive correlations are marked in red, negative in yellow. Clinical measures that were not applicable for HCs are in grey. P-values are not adjusted for multiple testing in this plot. *p<0.05, **p<0.01 and ***p<0.001.
ACE: acetate, PRO: propionate, BUT: butyrate, VAL: valerate, IVAL: isovalerate, IBUT: isobutyrate. HCAR2; hydroxycarboxylic acid receptor 2, FFAR2; free fatty acid receptor 2, BMI; body mass index, EDSS; expanded disability status scale, No. of Gd+; number of gadolinium enhancing lesions, AHR; aryl hydrocarbon receptor

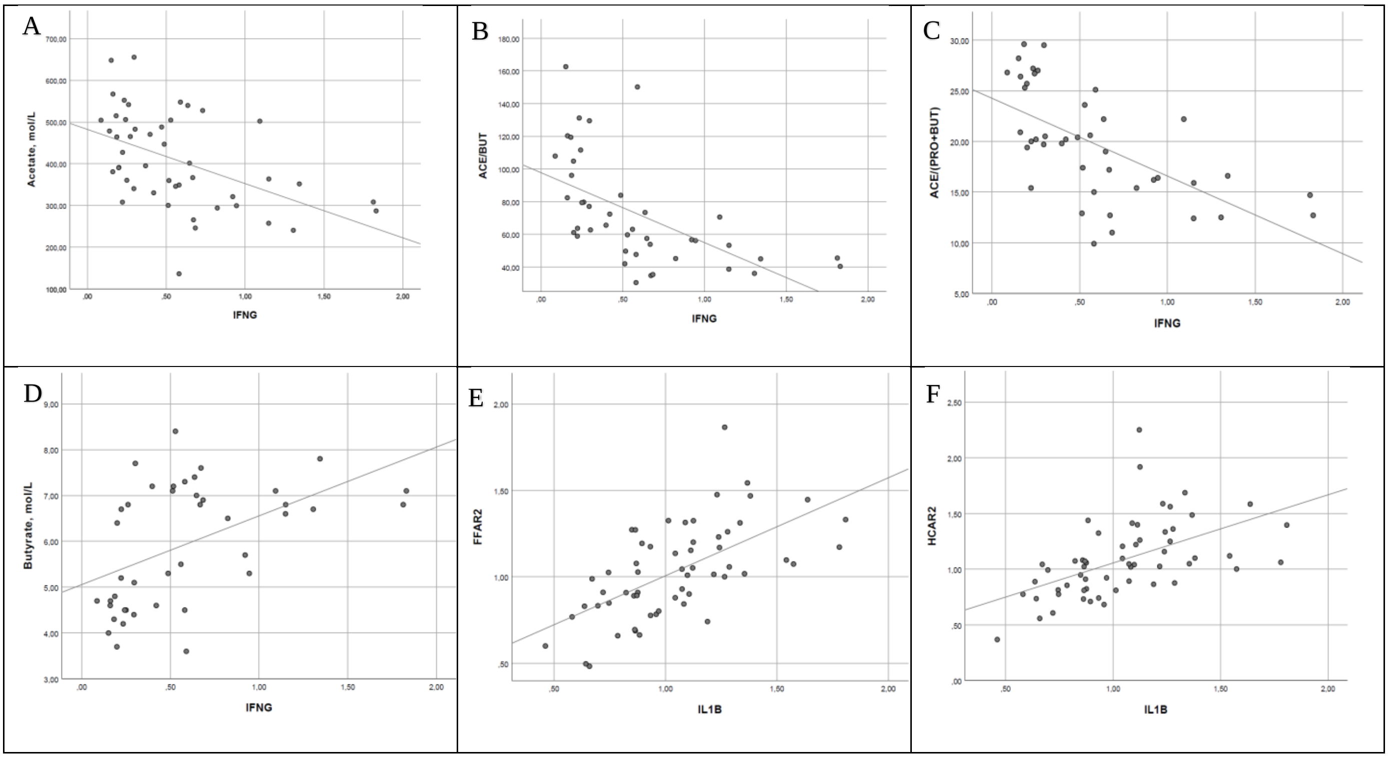

Figure S2. Scatter plots of selected correlations with q<0.01 and rho-values stronger than ± 0.5.
A) Acetate and *IFNG* in HCs (rho= -0.524, q<0.01). B) Acetate/butyrate and *IFNG* in HCs (rho= -0.689, q<0.001) C) acetate/(propionate + butyrate) and *IFNG* in HCs (rho= -0.735, q<0.001). D) Butyrate and *IFNG* in HCs (rho=0.559, q<0.001). E) FFAR2 and IL1B in MS patients (rho=0.638, q<0.0001). F) HCAR2 and *IL1B* in MS patients (rho=0.647, q<0.0001). Abbreviations: *IFNG:* Interferon-gamma, ACE: Acetate, BUT: Butyrate, PRO: Propionate, FFAR2: Free fatty acid receptor 2, HCAR2: hydroxycarboxylic acid receptor 2

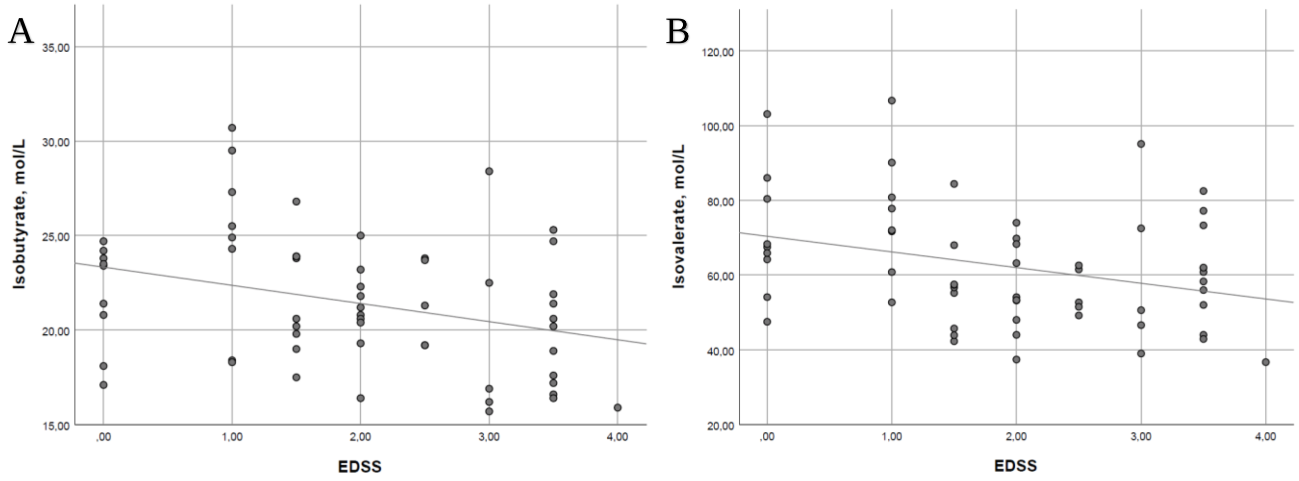

Figure S3. Scatter plots of clinical disability assessed by EDSS and the branched SCFAs. A) Isobutyrate and EDSS ((rho= -0.341, p=0.009). B) Isovalerate and EDSS (rho= -0.308, p=0.019). EDSS: Expanded Disability Status Scale

Workset... Options... Title: MS versus HC - SCFAs and biomarkers

Type: OPLS-DA Observations (N)=108, variables (K)=113 (X=111, Y=2)

Component	R2X	R2X(cum)	Eigenvalue	R2	R2(cum)	Q2	Limit	Q2(cum)	R2Y	R2Y(cum)	EigenvalueY	Significance
Model		0.118			0.614			0.221		1		
Predictive		0.0341			0.614			0.221		1		
P1	0.0341	0.0341	3.69	0.614	0.614	0.221	0.01	0.221	1	1	2	R1
Orthogonal in X(OPL...		0.0839			0							
O1	0.0839	0.0839	9.06	0	0							R1

Figure S4. Model window of the OPLS-DA model

Supplementary methods

Procedure of metabolomic analyses

Mass spectrometry-based methods were used to assess targeted metabolites at FIMM Metabolomics Unit, Institute for Molecular Medicine Finland. The final analysis for all metabolites were performed on an ACQUITY UPLC-MS/MS system (Waters Corporation, Milford, MA, USA). Chromatographic separation was done using 2.1 × 100 mm Acquity 1.7um BEH amide HILIC column (Waters Corporation, Milford, MA, USA), and temperature was maintained at 45°C. The total run time is 17.5 min including 2.5 min of equilibration step at a flow rate of 600 µL/min. Initially the gradient started with a 2.5 min isocratic step at 100% mobile phase B (ACN/ H_2O, 90/10 (v/v), 20 mM ammonium formate, pH at 3), and then rising to 100% mobile phase A (ACN/H_2O, 50/50 (v/v), 20 mM ammonium formate, pH at 3) over the next 10 min and maintained for 2min at 100% A and finally equilibrated to the initial conditions for 2.5 min. An injection volume of 5 µL of sample extract was used and two cycles of 300 µL of strong wash (methanol/isopropanol/ACN/H_2O, 25/25/25/25, 0.5% FA) and 900 µL of weak wash (methanol/isopropanol/ACN/H_2O, 25/25/25/25, 0.5% ammonium hydroxide) and in addition 2 min of seal wash (90/10, methanol/H_2O) were carried out. The auto-sampler was used to perform partial loop with needle overfill injections for the samples and standards and kept at 5 °C. The detection system, a Xevo® TQ-S tandem triple quadrupole mass spectrometer (Waters, Milford, MA, USA), was operated in both positive and negative polarities with a polarity switching time of 20 msec. Electro spray ionization (ESI) was chosen as the ionization mode with a capillary voltage at 0.72 and 3.70 KV in positive and negative polarities respectively. The source temperature and desolvation temperature of 150°C and 650°C, respectively, were maintained constantly throughout the experiment. Cone voltage and collision energy (CE) were

optimized for each compound. High pure nitrogen and argon gas were used as desolvation gas (600 L/hr) and collision gas (0.15 ml/min), respectively. Multiple Reaction Monitoring (MRM) acquisition mode was selected for quantification of metabolites with individual span time of 0.1 sec given in their individual MRM channels. The dwell time was calculated automatically by the software based on the region of the retention time window, number of MRM functions and the number of data points required to form the peak. MassLynx 4.1 software was used for data acquisition, data handling and instrument control. Data processing was done using TargetLynx software and metabolites were quantified by using labeled internal standards and external calibration curves.
